# Diabetes and gout: another role for SGLT2 inhibitors?

**DOI:** 10.1177/20420188241269178

**Published:** 2024-08-07

**Authors:** Clifford J. Bailey

**Affiliations:** College of Health and Life Sciences, Aston University, Gosta Green, Birmingham B4 7ET, UK

**Keywords:** diabetes, Gout, hyperuricaemia, sodium glucose co-transporter-2 inhibitors

## Gout and diabetes: Shared risks

People with gout are at increased risk of developing type 2 diabetes (T2DM) and people with T2DM have an increased risk of gout.^1–3^ For example, in a US National Health and Nutrition Examination Survey, diabetes was reported by 25.7% of people with gout compared with 7.8% of people without gout.^1^ In a retrospective cohort study of people with T2DM in the UK Clinical Practice Research Datalink, the risk of gout was increased by 48% compared with nondiabetic age- and sex-matched individuals.^2^ Increased gout in T2DM has been attributed mainly to coexistent morbidities, especially higher body mass index, reduced kidney function and increased hypertension.^2^ However, it is noted that chronic inflammation, insulin resistance and associated hyperinsulinaemia are features of both hyperuricaemic gout and T2DM, and may be pathogenic factors in common.^2,4–6^ Also, both hyperuricaemia and T2DM are recognised as independent risk factors for cardiovascular (CV) diseases and chronic kidney disease (CKD).^7–11^

## Treating hyperuricaemia

Hyperuricaemia in gout patients is customarily addressed by reducing dietary protein, reducing urate synthesis with xanthine oxidase inhibitors (e.g. allopurinol, febuxostat), increasing urate breakdown with a uricase (e.g. pegloticase) or increasing urate excretion with inhibitors of urate reabsorption by the kidney (e.g. probenecid, lesinurad, benzbromarone).^12–14^ Using these interventions, rapid reductions in serum urate can effectively reduce acute ‘flare-ups’ of joint pain caused by urate crystal precipitation. However, a sustained and substantial reduction of serum urate into the normal range appears to be required to reduce CV and renal risk, and multiple urate-lowering medicines may be required to achieve this, as well as other medications that specifically address CV and renal risk.^15–19^

## Sodium glucose co-transporter-2 inhibitors in T2DM

**Figure 1.**
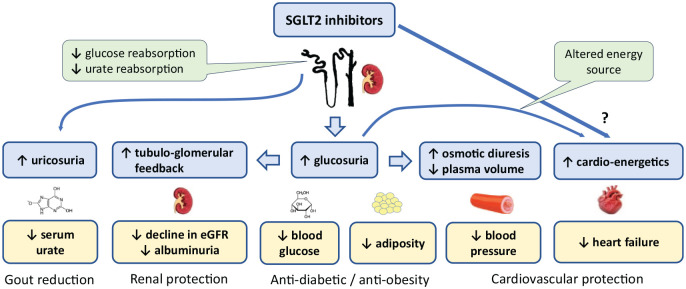
The diverse effects of SGLT2 inhibitors. In addition to anti-diabetic and anti-obesity effects, SGLT2 inhibitors offer cardio-protective and reno-protective effects as well as reducing serum urate concentrations. eGFR, estimated glomerular filtration rate; SGLT2, sodium glucose co-transporter-2.

Sodium glucose co-transporter-2 (SGLT2) inhibitors are now established blood glucose-lowering and body weight-lowering agents used in the treatment of T2DM.^20,21^ Their competitive inhibition of SGLT2 in the proximal renal tubules reduces the reabsorption of glucose from the renal filtrate. The resulting glucosuria accounts for the lowering of blood glucose, and the caloric loss enables the lowering of body weight (Figure 1). The reduced glucotoxicity and reduced adiposity then reduce insulin resistance and insulin concentrations.

Beyond their metabolic effects, SGLT2 inhibitors also offer cardioprotective and renoprotective properties.^22^ Typically, they reduce blood pressure in hypertensive individuals, attributed at least in part to the osmotic diuresis created by the glucosuria. The anti-hypertensive effect likely contributes to reductions in the onset and severity of heart failure by SGLT2 inhibitors (whether preserved or reduced ejection fraction): these effects are independent of blood glucose and body weight and are evident in people with or without T2DM.^23,24^ However, increased lipolysis in response to caloric deficit and reduced insulin enable SGLT2 inhibitors to improve cardiac energetics by increasing the availability and use of ketones and fatty acids by the myocardium (rather than glucose).^25,26^ Other effects of SGLT2 inhibitors on myocardial energy metabolism have been proposed,^27–30^ and beneficial effects of SGLT2 inhibitors on atherosclerotic vascular disease have been noted in some studies, linked with reductions in inflammation and oxidative stress.^31,32^

A consistent observation with SGLT2 inhibitor therapy is a slowing of the long-term decline in estimated glomerular filtration rate – thus improving the prognosis for CKD.^33–35^ This is seen in people with or without T2DM and exceeds that which could be attributed to reductions in blood glucose, body weight or blood pressure. It is also evident in people with normal or impaired renal function. The main mechanism appears to be increased tubuloglomerular feedback generated by SGLT2 inhibition which causes increased sodium to pass along the nephron. The increased sodium is sensed by cells of the macula densa, resulting in the formation of adenosine which constricts adjacent afferent glomerular arterioles. This in turn protects glomeruli by reducing intraglomerular pressure. SGLT2 inhibitors have also been reported to reduce renal inflammation and tubulointerstitial fibrosis, likely involving mechanisms additional to the inhibition of SGLT2.^35,36^

## SGLT2 inhibitors and gout

SGLT2 inhibitors have repeatedly reduced serum urate concentrations in clinical trials by ~35–45 μmol/L (~0.60–0.75 mg/dL) in people with T2DM with baseline values in the normal range (~200–400 μmol/L; ~3.3–6.7 mg/dL).^35,37^ The urate-lowering effect appears to be stronger in people with hyperuricaemia and symptomatic gout, typically exceeding 60 μmol/L (1.0 mg/dL).^38,39^ Treatment of T2DM with an SGLT2 inhibitor is associated with a lower incidence of gout by about 30% compared with treatment using a dipeptidyl peptidase-4 (DPP4) inhibitor or a glucagon-like peptide-1 (GLP-1) receptor agonist.^40,41^ Among people with T2DM and gout, the occurrence of gout ‘flare-ups’ is reduced in those treated with an SGLT2 inhibitor compared with use of a DPP4 inhibitor or GLP-1 receptor agonist, noting that many of these people are already receiving metformin plus anti-gout medication.^42–45^ Since these benefits are not closely correlated with the effects on glycaemic control or body weight, they have been attributed at least in part to the urate-lowering effect of SGLT2 inhibitors.^35,38^

The mechanism through which SGLT2 inhibitors lower serum urate is not yet fully established, but several studies have noted that the effect of SGLT2 inhibitors is additive to the urate-lowering effects of treatment with xanthine oxidase inhibitors and established uricosuric agents, indicating that SGLT2 inhibitors have a separate mode of action.^38^ Inhibition of SGLT2 in the proximal renal tubules increases the concentration of glucose remaining in the lumen of the tubules. This glucose can compete with urate at the GLUT9b glucose/urate transporter (SLC2A9), reducing urate reabsorption and increasing uricosuria.^35^ Added to this is the osmotic diuresis generated by glucosuria which could enhance the uricosuria.^46^

## Conclusion

Thus, having emerged as agents to manage T2DM, particularly in people who are overweight, SGLT2 inhibitors have recently expanded their indications to include heart failure and CKD for people with or without diabetes. Because the lowering of serum urate by SGLT2 inhibitors is independent of existing treatments for hyperuricaemia and additive to such treatments, SGLT2 inhibitors offer an extra resource for the management of hyperuricaemia and symptomatic gout. Moreover, the opportunity for improved long-term control of serum urate is anticipated to further reduce the risk of CV and renal disease associated with hyperuricaemia. Also, the independent cardiorenal protection provided by an SGLT2 inhibitor should afford additional health gains for individuals with hyperuricaemic gout.

## Declarations

**Ethics approval and consent to participate:** Not applicable.

**Consent for publication:** Not applicable.

**Availability of data and materials:** No new data included in this editorial.
